# Warming events projected to become more frequent and last longer across Antarctica

**DOI:** 10.1038/s41598-021-98619-z

**Published:** 2021-10-01

**Authors:** Sarah Feron, Raúl R. Cordero, Alessandro Damiani, Avni Malhotra, Gunther Seckmeyer, Pedro Llanillo

**Affiliations:** 1 University of Groningen, Leeuwarden, 8911 CE Netherlands; 2 Universidad de Santiago, Av. Bernardo O’Higgins 3363, Santiago, Chile; 3 Center for Environmental Remote Sensing, Chiba University, 1-33 Yayoicho, Inage Ward, Chiba, 263-8522 Japan; 4 University of Zurich, Winterthurerstrasse 190, 8057 Zürich, Switzerland; 5 Leibniz Universität Hannover, Herrenhauser Strasse 2, Hannover, Germany; 6 Alfred Wegener Institute (AWI), Am Handelshafen 12, 27570 Bremerhaven, Germany

**Keywords:** Natural hazards, Atmospheric science, Climate-change impacts

## Abstract

Summer temperatures are often above freezing along the Antarctic coastline, which makes ice shelves and coastal snowpacks vulnerable to warming events (understood as periods of consecutive days with warmer than usual conditions). Here, we project changes in the frequency, duration and amplitude of summertime warming events expected until end of century according to two emission scenarios. By using both global and regional climate models, we found that these events are expected to be more frequent and last longer, continent-wide. By end of century, the number of warming events is projected to double in most of West Antarctica and to triple in the vast interior of East Antarctica, even under a moderate-emission scenario. We also found that the expected rise of warming events in coastal areas surrounding the continent will likely lead to enhanced surface melt, which may pose a risk for the future stability of several Antarctic ice shelves.

## Introduction

**Figure 1:**
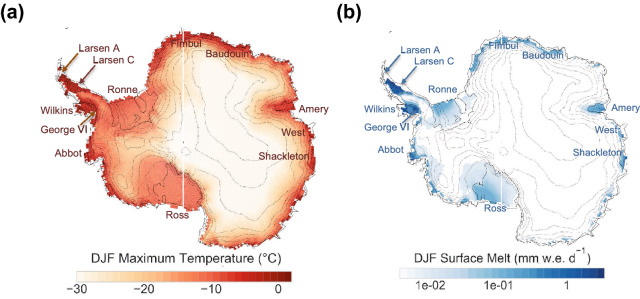
Summer temperatures along coastlines are frequently above 0 °C. (**a**) Mean daily DJF maximum air temperatures (TX). (**b**) Mean daily DJF surface melt (SM) intensities. Data over the period 1981–2010 were used (from the ERA5 dataset in case of the temperature and from the RACMO2 model in case of the surface melt). Plots were generated by using Python’s Matplotlib Library^44^.

The maximum air temperature (TX) in Antarctica is well below the freezing point in its vast interior but is much closer to 0 °C in summer (December–January–February (DJF)) on ice-shelves across the Antarctic ice sheets (Fig. 1a), especially at the northern tip of the Antarctic Peninsula^1^. While snow rarely melts in most parts of the continent, meltwater production is significant in coastal areas and on ice-shelves in the Antarctic Peninsula (Fig. 1b). The typical surface melt intensity on the Antarctic ice sheet in summer (up to 3 mm water equivalence (w.e.) per day) can be considered modest relative to the Greenland ice sheet^2^. Yet, these relatively modest seasonal averages mask occasionally high snow melt intensities triggered by warming events, also referred to as heatwaves (hereinafter referred to as HWs).

Temperatures associated with summertime warming events at high latitudes may be inconsequential elsewhere, but they can lead to widespread melt intensification along the Antarctic coastline. An intense and long HW affected the northern Antarctic Peninsula from February 5 to February 13, 2020. On February 6, 2020, thermometers at the Esperanza Base on reached 18.3 °C (the highest ever measured in the Antarctic continent)^3,4^. A combination of meteorological conditions led to this warming event. A ridge of high pressure was centered over Cape Horn at the beginning of the month, which allowed temperatures to rise. The Southern Hemisphere westerlies, that typically shield the Peninsula from warm air masses, were in a weakened state allowing extra-tropical warm air to cross the Southern Ocean^5^. Attributable to this single warming event, snowpack on Eagle Island (at the northern tip of the Antarctic Peninsula) melted 106 mm from February 6 to February 11^5^. About 20% of seasonal snow accumulation melted in this single event on Eagle Island^5^. While the conditions that lead to HWs are different elsewhere in Antarctica, these events are expected to become more frequent as temperatures rise.

Thirteen of the 17 stations (all having near‐continuous records of more than 30 years in length and spanning the different climatic regions of Antarctica) show a positive trend in their annual mean near-surface air temperature over the full length of their record^6^. The South Pole^7^ and the central West Antarctica^8^ have recorded since the late 1950s some of the largest increases in air temperature in the Southern hemisphere. The largest increases in air temperature in the continent have been recorded in Antarctic Peninsula^9,10^, resulting in strongly positive and statistically significant trends in the duration of melting conditions^11,12^. Yet, meltwater runoff contributions to the surface mass balance have been outpaced by the rise in snow accumulation over ice sheet interiors.

Although the surface mass balance for the total Antarctic Ice Sheet (including ice shelves) remains positive due to the increased snowfall^13,14^, glacier discharge in Antarctica is accelerating due to the weakening of ice shelves that have seen reduced their ability to restrain grounded ice-sheet flow^15–17^. In the Amundsen and Bellingshausen regions, ice shelf thinning arises mostly from basal melt attributable to intrusions of circumpolar deep water on the continental shelf^18–20^. However, in the Antarctic Peninsula, atmospheric warming and the resulting meltwater ponding also appears to be contributing to ice shelf weakening and accelerated glacier discharge into the ocean^21,22^.

Here we assess the progression and expected changes in summertime HWs understood as a period of at least 3 consecutive summer days with “very high” maximum temperatures (TX). We consider a TX value to be “very high” if it falls above the 90th percentile of the daily base climatology (built up by using daily TX values over a 30-year base period 1961–1990; see “Methods” section). Four HW metrics (HW duration, HW frequency, HW amplitude, and number of HWs per season) as well as TX90 (the number of summer days with “very high” maximum temperatures) were computed.

Our results are based on simulations under two representative concentration pathways (RCP4.5 and RCP8.5)^23^ from 15 Global Climate Models (GCMs) from the Coupled Model Inter-comparison Project Phase 5 (CMIP5)^24^ (Table S1). Although the TX90 estimates and HW metrics were computed for each GCM separately, our results are based on the multi model mean (MMM) of the HW metrics and the TX90 values. In order to compute the HW progression at specific points along the Antarctic coastline (Fig. S1), we also used simulations from a Regional Climate Model (RCM) from the Coordinated Regional Climate Downscaling Experiment (CORDEX)^25^: the Regional Atmospheric Climate Model RACMO, Version 2.1^26^, hereafter referred to as RACMO2. Temperature data from the ERA5 reanalysis^27^ were used for further comparisons.

## Results

**Figure 2:**
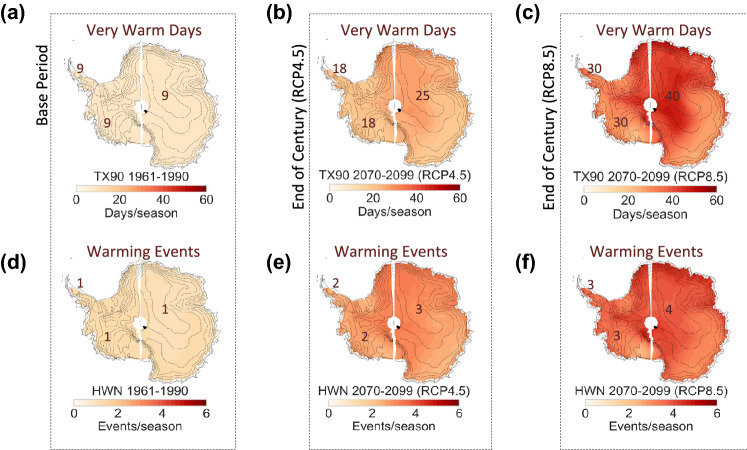
Summer Warming Events will become more frequent. Multi model mean (MMM) of summer HW metrics computed from 15 CMIP5 GCMs over the base period 1961–1990 (1st column), over the period 2070–2099 under the RCP4.5 scenario (2nd column), and over the period 2070–2099 under the RCP8.5 scenario (3rd column). (**a–c**) Number of very warm days per season (TX90); (**d–f**) number of HWs per season (HWN). Plots were generated by using Python’s Matplotlib Library^44^.

Our analysis suggests that across Antarctica, HWs will become more frequent and last longer, regardless of the emission scenario (Fig. 2). HW metrics (HW duration, HW frequency, and HW amplitude) as well as the number of very warm DJF days per season (TX90) are projected to significantly increase. Even under a moderate-emission scenario (RCP4.5). TX90 estimates in East Antarctica and along the coastline of Atlantic Ocean sector are projected to nearly triple (from 9 days per season in 1961–1990 to about 25 days per season in 2070–2099; first row in Fig. 2). Increments in very warm DJF days are relatively less pronounced in West Antarctica, including the Antarctic Peninsula. Very warm DJF days in Marie Byrd Land are expected to increase from 9 days per season in 1961–1990 to about 18 days per season in 2070–2099 (first row in Fig. 2).

Changes in the number of HW per season (HWN) exhibit a similar regional pattern. Under the RCP4.5 scenario, the number of HWs is projected to double (from 1 in 1961–1990 to about 3 in 2070–2099) in most of the West Antarctica, while they may triple at locations in the vast interior of East Antarctica (second row in Fig. 2). Projections under the RCP8.5 scenario are more striking but are consistent with those expected under the RCP4.5 scenario.

**Figure 3:**
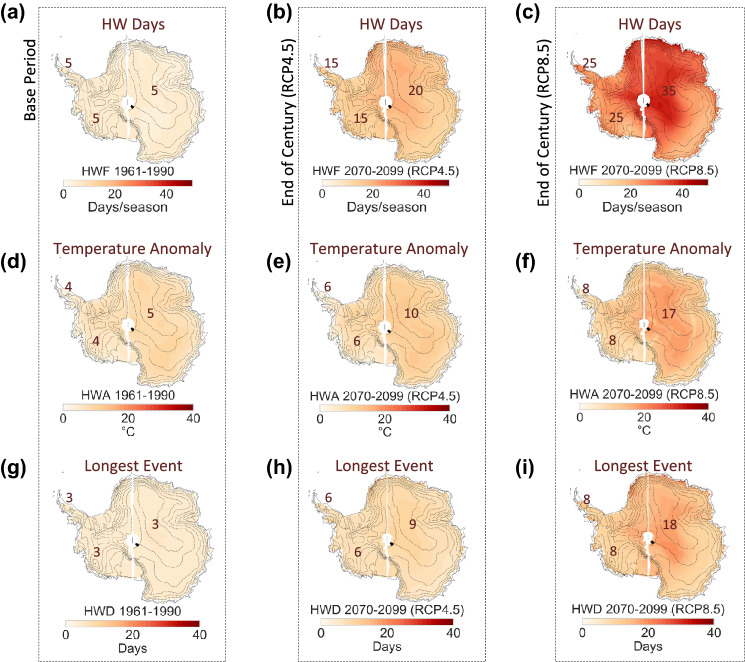
Summer Warming Events will become longer and more intense. Multi model mean (MMM) of summer HW metrics computed from 15 CMIP5 GCMs over the base period 1961–1990 (1st column), over the period 2070–2099 under the RCP4.5 scenario (2nd column), and over the period 2070–2099 under the RCP8.5 scenario (3rd column). (**a–c**) Number of HW days per season (HWF); (**d–f**) anomaly of the warmest day of the HWs (HWA); and (**g–i**) longest HW during a season (HWD). Plots were generated by using Python’s Matplotlib Library^44^.

HW days per season (HWF**)** are projected to rise from about 5 days in 1961–1990 to about 20 days in 2070–2099 (RCP4.5) in East Antarctica (first row in Fig. 3). HWF is projected to rise from about 5 days per season in 1961–1990 to about 15 days per season in 2070–2099 (RCP4.5) in West Antarctica and the Antarctic Peninsula (first row in Fig. 3). Under a moderate-emission scenario (RCP4.5), the temperature anomaly of the warmest day of the HWs (HWA) is expected to increase by about 5 °C by end of century (2070–2099) throughout East Antarctica, whereas HWA estimates in West Antarctica and the Antarctic Peninsula are expected to remain below 10 °C (second row in Fig. 3).

The length in days of the longest HW during a season (HWD; in days) is projected to surge in the vast interior of East Antarctica from less than 5 days in 1961–1990 to about 9 days in 2070–2099 (RCP4.5) (third row in Fig. 3). HWD estimates in West Antarctica and the Antarctic Peninsula are expected to nearly double by end of century even under a moderate-emission scenario (RCP4.5). Although the HWD increments are less pronounced in West Antarctica and the Antarctica Peninsula, changes in these regions are more consequential in term of surface melt than those occurring in East Antarctica.

Our projections show significant increases in HWs and “very warm” DJF days in the vast interior of Antarctica. However, since the temperatures in these regions are currently well below the freezing point (Fig. 1a), it is unlikely that HWs in the interior of the continent will lead to widespread surface melt during this century, even under a high-emission scenario (RCP8.5). In West Antarctica and in coastal areas surrounding the continent, where the maximum DJF temperature is already much closer to 0 °C (Fig. 1a), HWs currently occurring do lead to intense surface melt.

In order to investigate the effects of the expected increase in “very warm” temperatures on melt intensification at specific coastal locations in Antarctica, we used high-resolution data from RACMO2 (48-km grid resolution). The RACMO2 model has been broadly evaluated across Antarctica. Both its simulations of the air temperature^28^ and meltwater production^11,29–31^ have been extensively tested. In particular, we focused on the grid points that exhibit the highest surface melt on several major ice shelves (Fig. S1).

**Figure 4:**
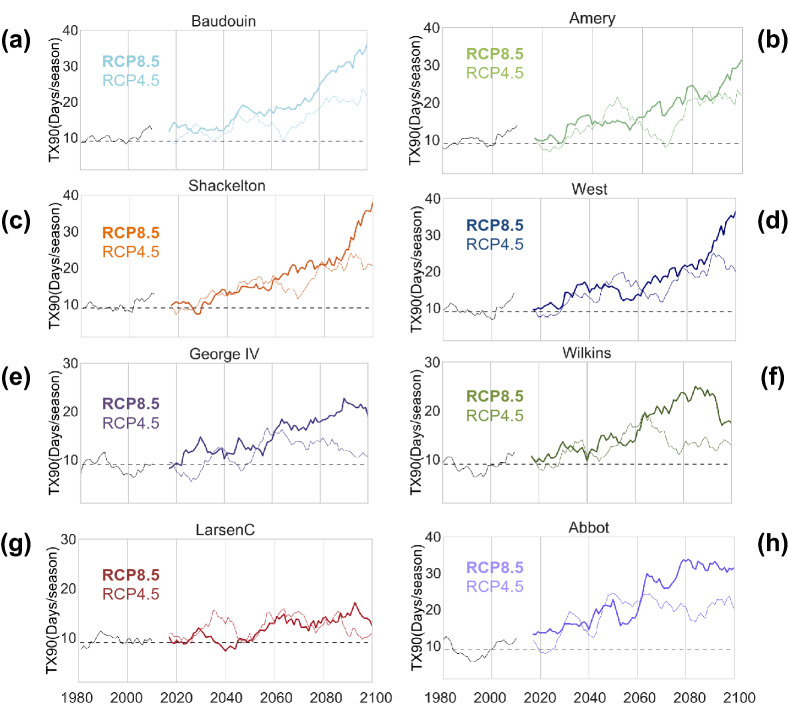
Summer very warm days will surge on most of the Antarctic Ice shelves. 10-year centered moving averages of “very warm” DJF days (TX90) expected under the RCP4.5 scenario (thin lines) and under the RCP8.5 scenario (bold lines) for selected ice shelves: Baudouin (**a**), Amery (**b**), Shackleton (**c**), West (**d**), George VI (**e**), Wilkins (**f**), Larsen C (**g**) and Abbot (**h**). Time series corresponding to Ross, Ronne, and Amery are shown in the Supplementary Information. RACMO2 simulations were used (see “Methods” section). Plots were generated by using Python’s Matplotlib Library^44^.

Projections of “very warm” DJF days (TX90) until end of century show significant inter-decadal increments for several Antarctic ice shelves, especially beyond the Antarctic Peninsula (Fig. 4, Supplementary Fig. S2). The greatest increments in “very warm” DJF days are expected to occur on Abbot, West, Shackelton, Fimbul and Baudouin ice shelves, where the TX90 estimates may double on average (relative to the base period level) even under a moderate-emission scenario. Under a high-emission scenario (RCP8.5), more than one third of the summer days may become “very warm” by end of century on West and Shackelton ice shelves and Baudouin and Fimbul ice shelves. The projected rise in “very warm” DJF days is somehow less pronounced in the case of ice shelves in the Antarctic Peninsula (Larsen C, George IV and Wilkins) as well as in the case of major ice shelves such as Ross and Ronne (Fig. S2). Yet, we found that, under the RCP8.5 scenario, “very warm” DJF days are expected to double by end of century on George IV and Wilkins ice shelves and to increase by about 50% on the northern most section of the Larsen C ice shelf. The surge in the number of “very warm” DJF days expected on ice shelves around Antarctica will lead to significant increases in surface melt and meltwater production.

Surface melt is strongly responsive to increases in air temperature. Responding to very high temperatures the daily surface melt intensity on Antarctic ice shelves can often double the seasonal mean. Changes in the DJF surface melt simulations are consistent with the changes in DJF air temperatures, but the response of the surface melt to increases in air temperature is nonlinear (Figs. S3, S4). For example, responding to very warm temperatures, the daily surface melt intensity on the Larsen A ice shelf nearly quintupled (relative to the seasonal mean) prior its collapse in January 1995 (pre-collapse record monthly averages of the daily surface melt intensities on the Larsen A ice shelf are also shown in Fig. S3b). Meltwater production on Larsen B also exceeded historical records before collapsing in February–March 2002^32^.

The short-term surface melt events associated with HWs on ice shelves may not significantly contribute to the annual Antarctic mass losses (volume change) because in Antarctica most of the annual meltwater from snowmelt refreezes^33^. Snow properties change in case of melting much more than in case of meltwater refreezing. However, surface melt does alter the surface radiative budget by changing the surface albedo. The refreezing of meltwater leads to an increase in snow grain size, which in turn decreases the albedo^34^. Moreover, periods of consecutive very warm days (i.e., warming events or heat waves) can lead to persistent meltwater ponds. For example, in January 2020 (at the peak of the 2019–2020 melt season), meltponds spanned a vast area of the George VI ice shelf spanning a length of about 140 km on the shelf. The impressive extent of the melt suggests that it resulted from multiple days of melting^35^. Melt ponds are darker than surrounding ice and therefore have a lower albedo, thus absorbing more shortwave radiation in a feedback loop that can lead to further and rapid melting^36^. On an ice shelf, ponded water can fill and magnify ice crevasses, destabilizing and eventually breaking the ice shelf up^37,38^. This process, often referred to as hydrofracturing, was the leading mechanism in the collapse of the Larsen A and Larsen B ice shelves^39–41^.

The extreme surface melt intensities on the Larsen A ice shelf prior its collapse led to record levels of meltwater production, which probably contributed to the disintegration of the ice shelf via meltwater ponding and hydrofracture. As shown in Fig. S3b, the daily surface melt intensity on Larsen A was about 12.7 mm w.e. day^−1^ in December 1994 and further increased to 19.6 mm w.e. day^−1^ in January 1995. The resulting meltwater production reached a record of about 1000 mm w.e. (12.7 × 31 days + 19.6 × 31 days) over that short period (December 1994–January 1995).

The record pre-collapse meltwater production (1000 mm w.e.) on Larsen A has not been observed on other ice shelves elsewhere in Antarctica. However, the rise in frequency of HWs and the resulting surface melt may produce such extreme values of meltwater production on several major ice shelves in the Antarctic Peninsula before end of century. Although we cannot project how specific ice shelves will actually respond, levels of meltwater production as those observed on Larsen A before its collapse (1000 mm w.e.) pose a risk for the stability of any ice shelf. Several paths can lead an ice shelf to reaching the record 1000 mm w.e. meltwater production; for example, an intense warming event (i.e., a relatively short stream of consecutive days with extreme temperatures), or a long warm summer (i.e., a summer with a relatively high number of melt days), or a combination of both (as actually occurred in the case of Larsen A and B before collapsing).

**Figure 5:**
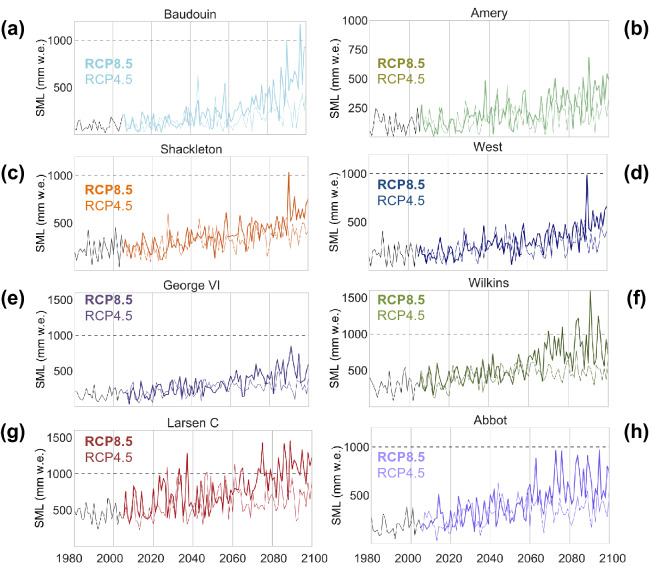
Meltwater production will significantly increase along the Antarctic coastline. Time series of DJF water production (SML) expected under the RCP4.5 scenario (thin lines) and under the RCP8.5 scenario (bold lines) for selected ice shelves: Baudouin (**a**), Amery (**b**), Shackleton (**c**), West (**d**), George VI (**e**), Wilkins (**f**), Larsen C (**g**) and Abbot (**h**). Time series corresponding to Ross, Ronne, and Amery are shown in the Supplementary Information. RACMO2 simulations were used (see “Methods” section). Plots were generated by using Python’s Matplotlib Library^44^.

The more frequent “very warm” days will enhance meltwater production per season (SML). As in the case of the “very warm” DJF days (Fig. 4, Supplementary Fig. S2), a surge is projected in SML values until end of century for several Antarctic ice shelves (Fig. 5, Supplementary Fig. S5). In absolute terms, major increments are projected to occur on Larsen C, Abbot, Shackelton, and Baudouin ice shelves, where the DJF meltwater production can increase on average (relative to the base period level) by about 200 mm w.e. per season under a moderate-emission scenario (Fig. 5). Under a high-emission scenario (RCP8.5), by end of century, sections of the George IV, West, Fimbul and Amery ice shelves are projected to reach meltwater production levels (about 300 mm w.e. per season) that are on average currently observed only in the Antarctic Peninsula. Fortunately, the projected rise in the meltwater production is less pronounced in the case of major ice shelves such as Ross and Ronne (Fig. S5). RACMO2 simulations (Fig. S6) confirm that, with the exception of the northern most part of Larsen C, the multi-decadal average of the meltwater production of the Antarctic ice shelves is expected to remain well below the risky 1000 mm w.e. level (the meltwater produced by Larsen A and Larsen B right before its collapse).

Although decadal averages of the DJF meltwater production will likely remain moderate on most of the Antarctic ice shelves, Fig. 5 shows that there is an increasing likelihood of reaching risky levels (1000 mm w.e.) during a given season on several ice shelves. Figure 5 also shows a significant surge in the inter-annual variability of the DJF meltwater production. According to RAMCO2 projections, the multi-decadal variability (taken as the standard deviation of annual SML estimates) is projected to double from 1961–1990 to 2070–2099. Attributable to the enhanced inter-annual variability (associated with more frequent and intense HWs), the DJF meltwater production may occasionally surpass the risky 1000 mm w.e. level. This is the case of the northern most section of Larsen C, which is at risk of reaching the threshold by mid-century even under a moderate-emission scenario (Fig. 5g). Before the end of century, Wilkins, Shackleton, Abbot and Baudouin ice shelves may also reach, at least during one season, the risky 1000 mm w.e. level (although only under the RCP8.5 scenario).

## Discussion

Antarctic HWs are expected to be more frequent and last longer, continent-wide. By end of century, the number of warming events is projected to double in West Antarctica (including the Antarctic Peninsula) and to triple in the vast interior of East Antarctica, even under a moderate-emission scenario. The length in days of the longest event during a season is projected to double in West Antarctica and the Antarctica Peninsula up to about 6 days by end of century. Projections under a high-emission scenario are even more striking.

The increases in warming events in coastal areas surrounding the continent (where the DJF temperature is already close to 0 °C) are particularly concerning since they can lead to enhanced surface melt. Surface melt responds to increases in air temperature through a nonlinear relationship. Nonlinearity arises from the melt-albedo feedback and from the effect of persistent warm days (surface melt of ice shelves/sheets already undergoing surface melt results in sharp mass losses)^32^. Antarctica is therefore particularly sensitive to rapid increases in melting and mass loss in response to relatively small increases in air temperature.

In response to rapidly warming air temperatures, surface melt is projected to significantly increase in upcoming decades regardless of the emission scenario. Sustained high temperatures, significantly above freezing, will become more frequent and surface melt intensities are expected to approach those currently observed in the Antarctic Peninsula elsewhere in Antarctica. Nevertheless, the impact of warming events and the associated enhanced surface melt is not expected to become large in terms of long-term ice-sheet mass balance (volume change) because they are constrained mostly to coastal areas and because of refreezing.

Most of the meltwater from snowmelt currently refreezes in Antarctica. However, the projected surge in the number of consecutive very warm days (i.e., the warming events duration) will lead to lasting and larger meltwater ponds. On an ice shelf, ponded water can fill and magnify ice crevasses, destabilizing and eventually breaking the ice shelf up via hydrofracturing. Surface meltwater ponding may drive the collapse of Larsen A and Larsen B ice shelves as well as the speedup of tributary glaciers in the northeast Antarctic Peninsula. Potential ice-shelf disintegration and subsequent increases in ice sheet mass loss highlight the importance of ice shelf stability.

Attributable to the projected rise in warming events and very warm days, we found that several Antarctic ice shelves have an increasing probability of reaching the record meltwater production that the Larsen A and Larsen B ice shelves exhibited before their collapse. For some ice shelves (Larsen C, Wilking and Abbot), the risk of surpassing the risky 1000 mm w.e. level is considerable well before end of century. Although, we do not aim to project how specific ice shelves will actually respond, levels of meltwater production as those observed on Larsen A before its collapse (1000 mm w.e.) likely pose a risk for the stability of any ice shelf. The projected warming events and the associated enhanced surface melt, especially after mid-century under the RCP8.5 scenario, raise concerns about the stability of several ice shelves, which may have important consequences in terms of ice shelf buttressing and sea level rise.

Lastly, we found substantial differences between warming figures under different emission scenarios (RCP8.5 and RCP4.5), especially in the second half of this century. Therefore, curbing greenhouse gas emissions and limiting global warming will make a significant difference in the number of extreme warming events that the Antarctic ice shelves will have to endure.

## Methods

We applied a widely used methodology for assessing changes in the occurrence probability of extreme events^42,43^. For each DJF day and at each location (or grid point of the climate model) and, we used a 15-day rolling window of the daily estimates of the maximum air temperature (TX) over a base period of 30 years (1961–1990), in order to form datasets of 450 values (15 days × 30 years). Then, we used these datasets of 450 values in order to compute the mean (that defined the daily base climatology) and the daily 90th percentile threshold. For each summer season and at each location (or grid point of the climate model), we took the number of “very warm” DJF days (TX90) as equal to the number of DJF days when the maximum temperature (TX) exceeded the 90th percentile threshold.

Here, we considered a HW as period of at least 3 consecutive “very warm” DJF days. According to this definition, for each summer (DJF) season and at each location (or grid point of a climate model), we computed the following HW metrics:

HWA: the HW amplitude (i.e., TX anomaly of the warmest day of any HW during a season);
HWD: the HW duration (i.e., the length in days of the longest HW during a season);
HWF: the HW frequency (i.e., the number of HW days per season); and the
HWN: the number of HWs per season.

Simulations for TX90 estimates and HW metrics were conducted under two representative concentration pathways (RCP4.5 and RCP8.5)^23^ from 15 CMIP5 GCMs (see Table S1). Results corresponding to the MMM of HW metrics and TX90 estimates were first computed for each GCM separately. In order to avoid assigning more weight to one model or ensemble over another, all CMIP5 GCMs that had available daily TX estimates and monthly estimates of the surface melt were used. Although we selected only the first ensemble member of each available model, we confirmed that selecting a different ensemble makes no considerable difference in the MMM.

Projections of HW metrics (and meltwater production) were also computed by using simulations from a Regional Climate Model (RCM) from the Coordinated Regional Climate Downscaling Experiment (CORDEX)^25^: the Regional Atmospheric Climate Model RACMO, Version 2.1 (48-km grid resolution)^26^, here referred to as RACMO2. RACMO2 has been broadly evaluated across Antarctica. Its simulations of the air temperature^28^ and meltwater production^11,29–31^ have been extensively tested and are considered to be reliable.

Figure S7 allows comparing simulations from GCMs and RACMO2. It shows both the MMM and the Multi model standard deviation (MMSD) of very warm DJF days per season (TX90) (see first and second row in Fig. S7, respectively). The MMSD provides a metric of the spread of HW metrics from different models. Although MMSD values in Fig. S7 confirm the sizable intermodel differences, we should underline the fact that the 15 models did agree on the direction of the changes; all of the models project more frequent and longer HWs across Antarctica. RACMO2 simulations (third row in Fig. S7) not only agree on the direction of the expected changes but also match the GCM MMM remarkably well (first row in Fig. S7). As shown in Fig. S7, despite the coarser resolution of GCMs, simulations from both regional and global models exhibited similar regional features; the relatively small differences between simulations from RACMO2 and from GCMs (MMM) in Fig. S7, are all within the bounds defined by the MMSD. RACMO2 simulations have even been used in prior efforts^32^ as a reference for testing CMIP5 GCMs.

The higher resolution of RACMO2, enabled us to focus on points of interest along the Antarctic coastline (Fig. S1). Antarctic ice shelves amount to tens of thousands of square kilometers. Accordingly, surface melt intensity on an ice shelf generally exhibits significant geographical differences. Here, we focus on the grid points on each ice shelf that exhibited the highest surface melt over the period 1981–2010 according to the RACMO2 model. These grid points are shown in Fig. S1 while their coordinates are shown in Table S2; the surface of these grid points is about one thousand square kilometers.

For the grid points shown in Fig. S1, we computed the time series of the number of very warm days per season (TX90; Fig. 4), and also, by using the daily estimates of the surface melt (SM), we computed the meltwater production per season (SML; Fig. 5).

## Supplementary Information

Supplementary Information.

## Data Availability

Data from the Global Climate Models (GCMs) were obtained from the World Climate Research Programme’s Working Group for the Coupled Model Intercomparison Project—Phase 5: https://esgf-node.llnl.gov/. RAMCO2 simulations under the Coordinated Regional Climate Downscaling Experiment (CORDEX) are available from the Earth System Grid Federation (ESGF): https://esgf-data.dkrz.de/projects/cordex-dkrz/. The code generated during this study is available from the corresponding author.
