# Supplementary material for: Warming events projected to become more frequent and last longer across Antarctica
Source: Sci Rep. 2021 Oct 1;11:19564. doi: 10.1038/s41598-021-98619-z (PMC8486840; doi:10.1038/s41598-021-98619-z)
Supplement: Supplementary file 1 — Supplementary Information. [file 41598_2021_98619_MOESM1_ESM.pdf]

Supplementary Information

Warming events projected to become more frequent and last longer across Antarctica

Sarah Feron[1,2,*], Raúl R. Cordero[2,*], Alessandro Damiani[3], Avni Malhotra[4], Gunther Seckmeyer[5], Pedro Llanillo[6]

1 University of Groningen, Wirdumerdijk 34, 8911 CE, Leeuwarden, Netherlands
2 Universidad de Santiago, Av. Bernardo O'Higgins 3363, Santiago, Chile
3 Center for Environmental Remote Sensing, Chiba University, 1-33 Yayoicho, Inage Ward, Chiba, 263-8522, Japan
4 University of Zurich, Winterthurerstrasse 190, 8057 Zürich, Switzerland
5 Leibniz Universität Hannover, Herrenhauser Strasse 2, Hannover, Germany
6 Alfred Wegener Institute (AWI), Am Handelshafen 12, 27570 Bremerhaven, Germany

* Corresponding Authors
Sarah Feron
s.c.feron@rug.nl
Raúl R. Cordero
raul.cordero@usach.cl

Additional Methodological Details

1) Calculation of TX90 estimates and HW metrics

In order to compute the number of "very warm" DJF days (referred to as TX90) and the HW metrics, we applied a widely used methodology for assessing changes in the occurrence probability of extreme events[42,43]. This methodology involved the following steps:

- For each DJF day and at each location (or grid point of the climate model) and, we used a 15-day rolling window of the daily estimates of the maximum air temperature (TX) over a base period of 30 years (1961-1990), in order to form datasets of 450 values (15 days x 30 years).

- For each DJF day and at each location (or grid point of the climate model), we used the corresponding datasets of 450 values (15 days x 30 years) in order to compute the mean (that defined the daily base climatology) and the daily 90th percentile (that we used as a threshold for defining "very warm" DJF days).

- For each summer (DJF) season and at each location (or grid point of the climate model), we took the number of "very warm" DJF days (TX90) as equal to the number of DJF days when the maximum air temperature (TX) exceeded the 90th percentile threshold. By definition, TX90 estimates tend to be around 9 days for summer (DJF) seasons over the base period but, as we have shown, they may exhibit significant changes in the future.

- For each summer (DJF) season and at each location (or grid point of the climate model), we computed the following HW metrics:

> The number of HWs per season (HWN). We considered a HW as a period of at least 3 consecutive "very warm" days (i.e. days when the TX value exceeded the corresponding 90th percentile).
>
> The HW amplitude (HWA), which we took as equal to the TX anomaly of the warmest day of any HW during a season. The anomalies were taken as equal to the departure of daily TX estimate from the daily base climatology;
>
> The HW duration (HWD), which we took as equal to the length in days of the longest HW during a season;
>
> The HW frequency (HWF), which we took as equal to the number of HW days per season).

2) Multi Model Mean (MMM)

TX90 projections and HW metrics shown above are based on simulations under two representative concentration pathways (RCP4.5 and RCP8.5)[23] from 15 Global Climates Models (GCMs) from the Coupled Model Inter-comparison Project Phase 5 (CMIP5[24]; see Table S1). Although the TX90 estimates and HW metrics were computed for each GCM separately, our results are based on MMM, which was computed by simply averaging TX90 estimates rendered by each model.

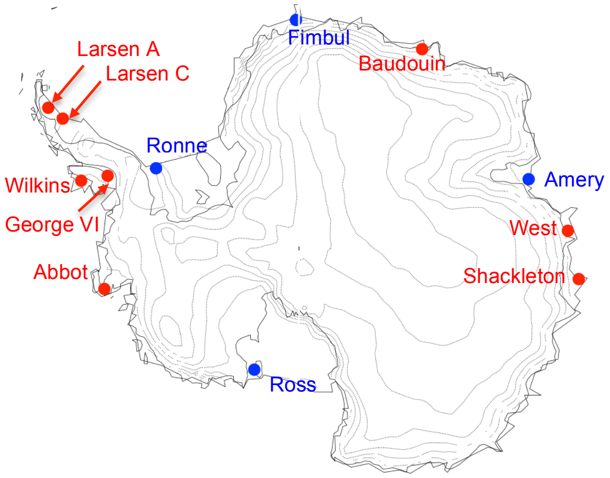

Fig. S1.
Grid points of the RACMO2 model that exhibited on each ice shelf the highest surface melt over the period 1981-2010. The surface of each of these grid points is about one thousand square kilometers; coordinates are shown in Table S2 Major ice shelves were divided into two groups; those that exhibited a relatively high mean surface melt (red points) and those exhibited a relatively low mean surface melt (blue points). Plot was generated by using Python's Matplotlib Library[44].

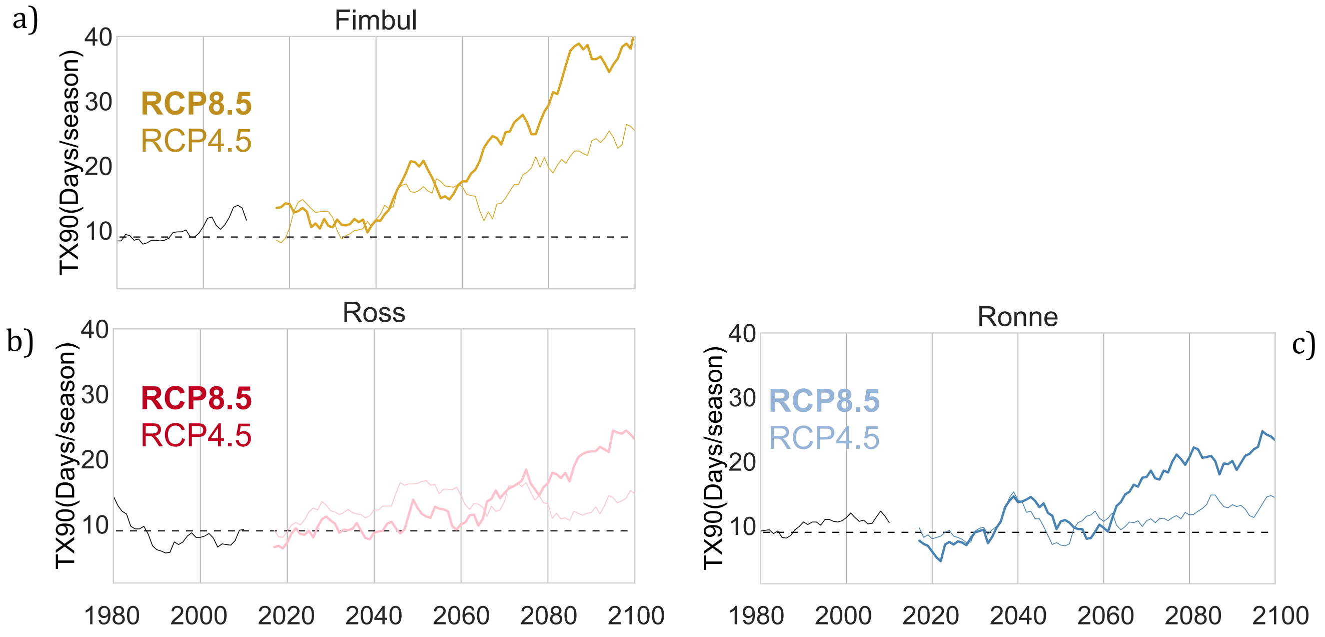

Fig. S2
10-year centered moving averages of the "very warm" DJF days (TX90) expected under the RCP4.5 scenario (thin lines) and under the RCP8.5 scenario (bold lines) for the following ice shelves: Fimbul (a), Ross (b) and Ronne (c). RACMO2 simulations were used (see Methods). Plots were generated by using Python's Matplotlib Library[44].

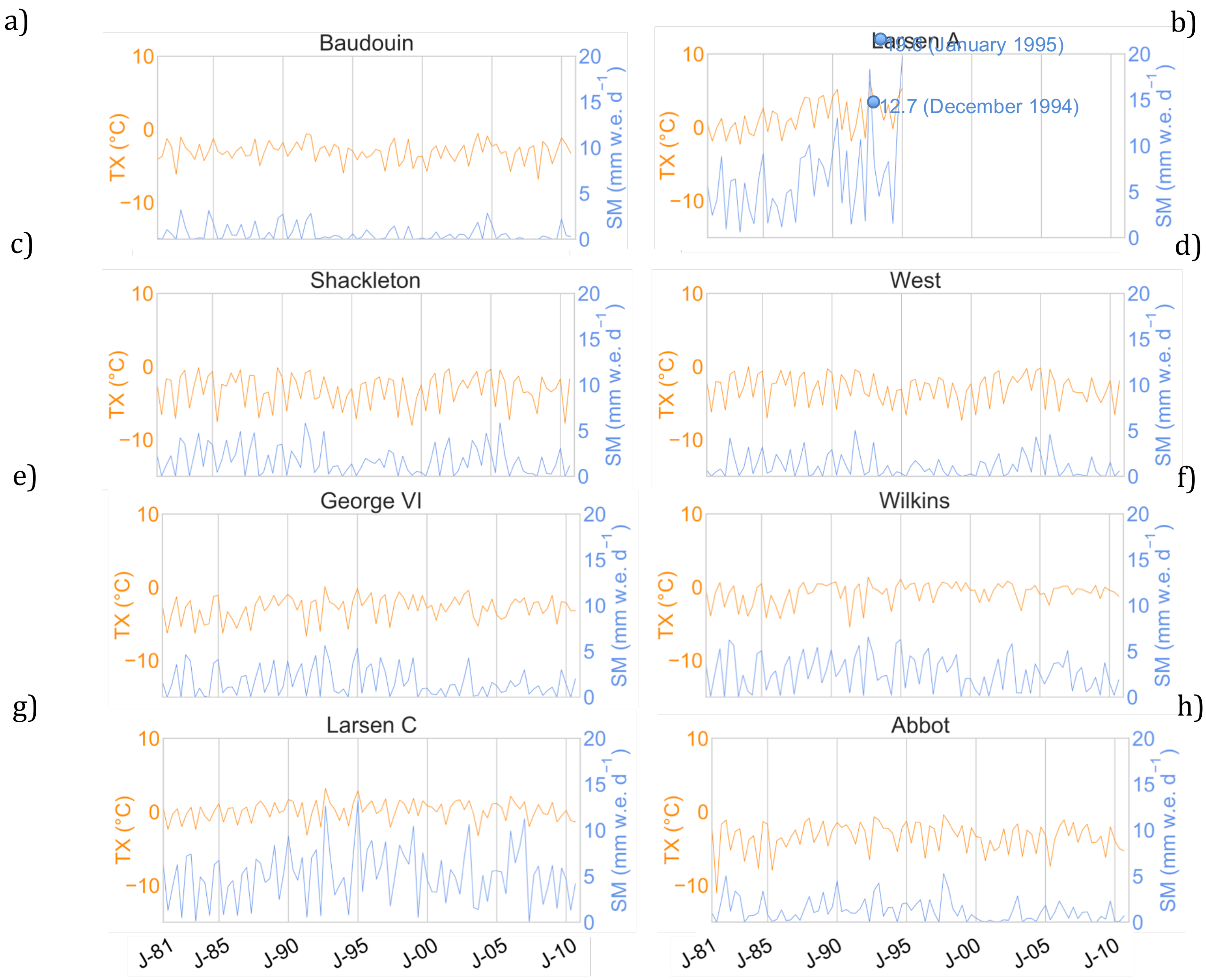

Fig. S3. Monthly means of the daily DJF maximum temperature and of the daily DJF surface melt (SM) intensities for the following ice shelves: Baudouin (a), Larsen A (b), Shackleton (c), West (d), George VI (e), Wilkins (f), Larsen C (g) and Abbot (h). In the case of the Larsen A ice shelf (b), pre-collapse monthly averages of the daily DJF surface melt intensities (December 1994 and January 1995) are indicated in the plot. Data from the ERA5 dataset were used in the case of the temperature while RACMO2 simulations were used in the case of the surface melt.

Plots were generated by using Python's Matplotlib Library[44].

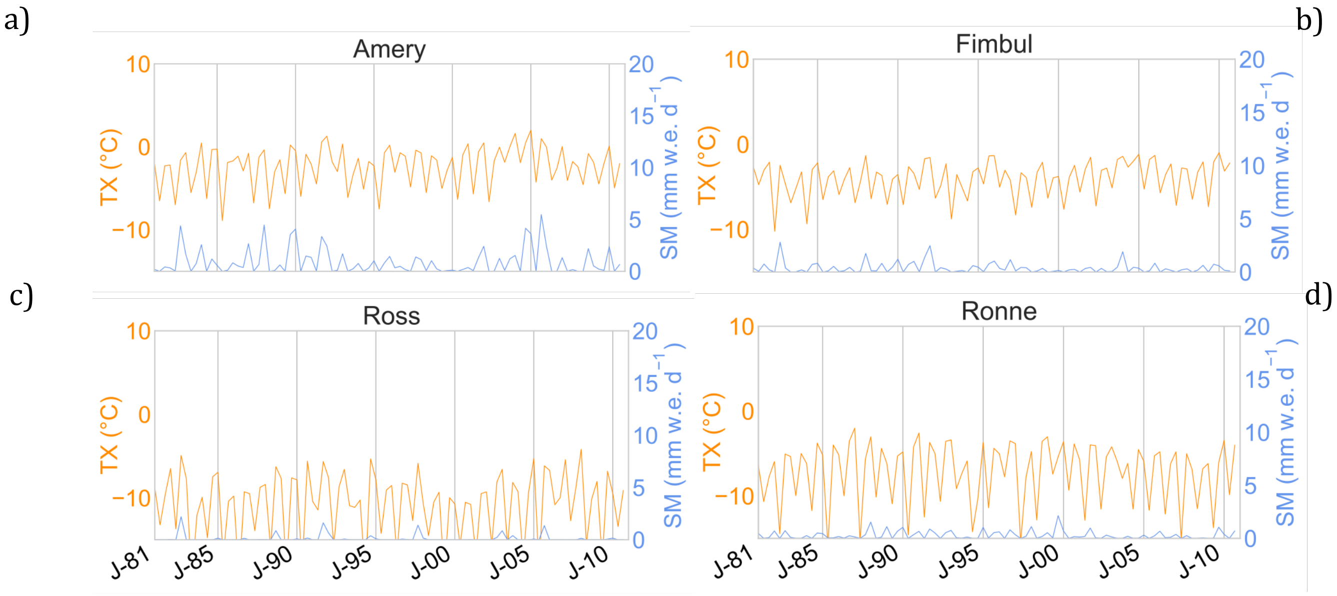

Fig. S4. Monthly means of the daily DJF maximum temperatures (TX) and of the daily DJF surface melt (SM) intensities for the following ice shelves: Amery (a), Fimbul (b), Ross (c), and Ronne (d). Data from the ERA5 dataset were used in the case of the temperature while simulations of RACMO2 were used in the case of the surface melt. Plots were generated by using Python's Matplotlib Library[44].

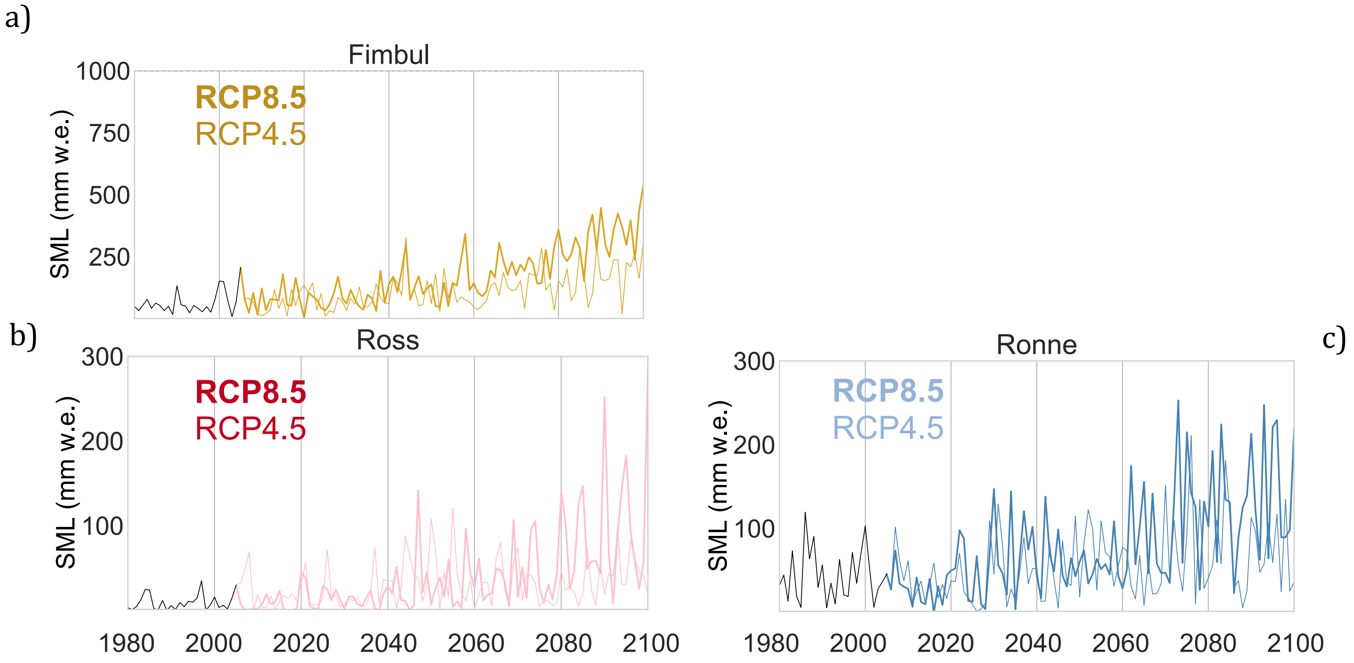

Fig. S5
Time series of DJF water production under the RCP4.5 scenario (thin lines) and under the RCP8.5 scenario (bold lines) for the following ice shelves: Fimbul (a), Ross (b) and Ronne (c). RACMO2 simulations over the historical period (1980-2005) and over the period 2006-2100 were used. Plots were generated by using Python's Matplotlib Library[44].

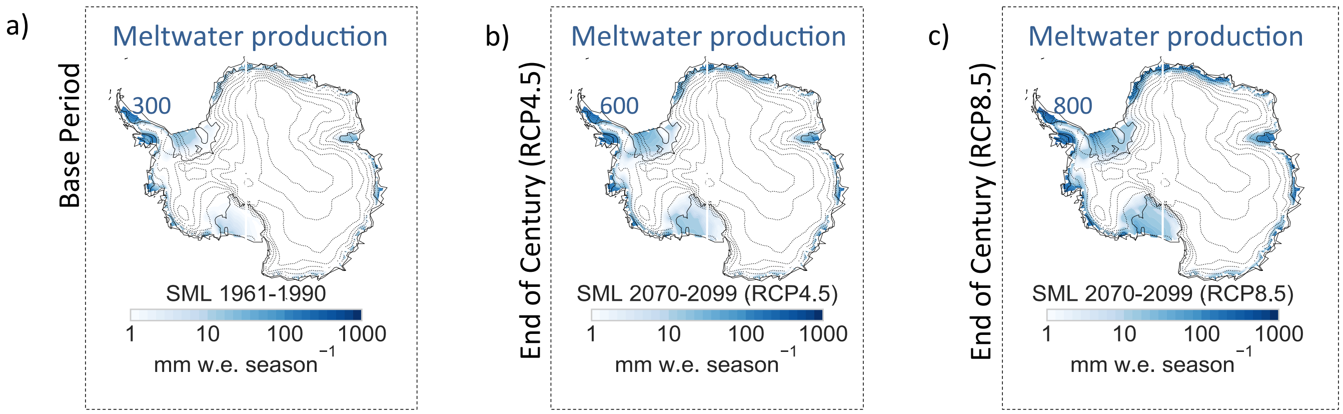

Fig. S6.
a-c) Mean of the total DJF meltwater production computed by using RACMO2 simulations. Means were computed over the base period 1961-1990 (a), over the period 2070-2099 under the RCP4.5 scenario (b), and over the period 2070-2099 under the RCP8.5 scenario (c). Plots were generated by using Python's Matplotlib Library[44].

GCMs versus RACMO

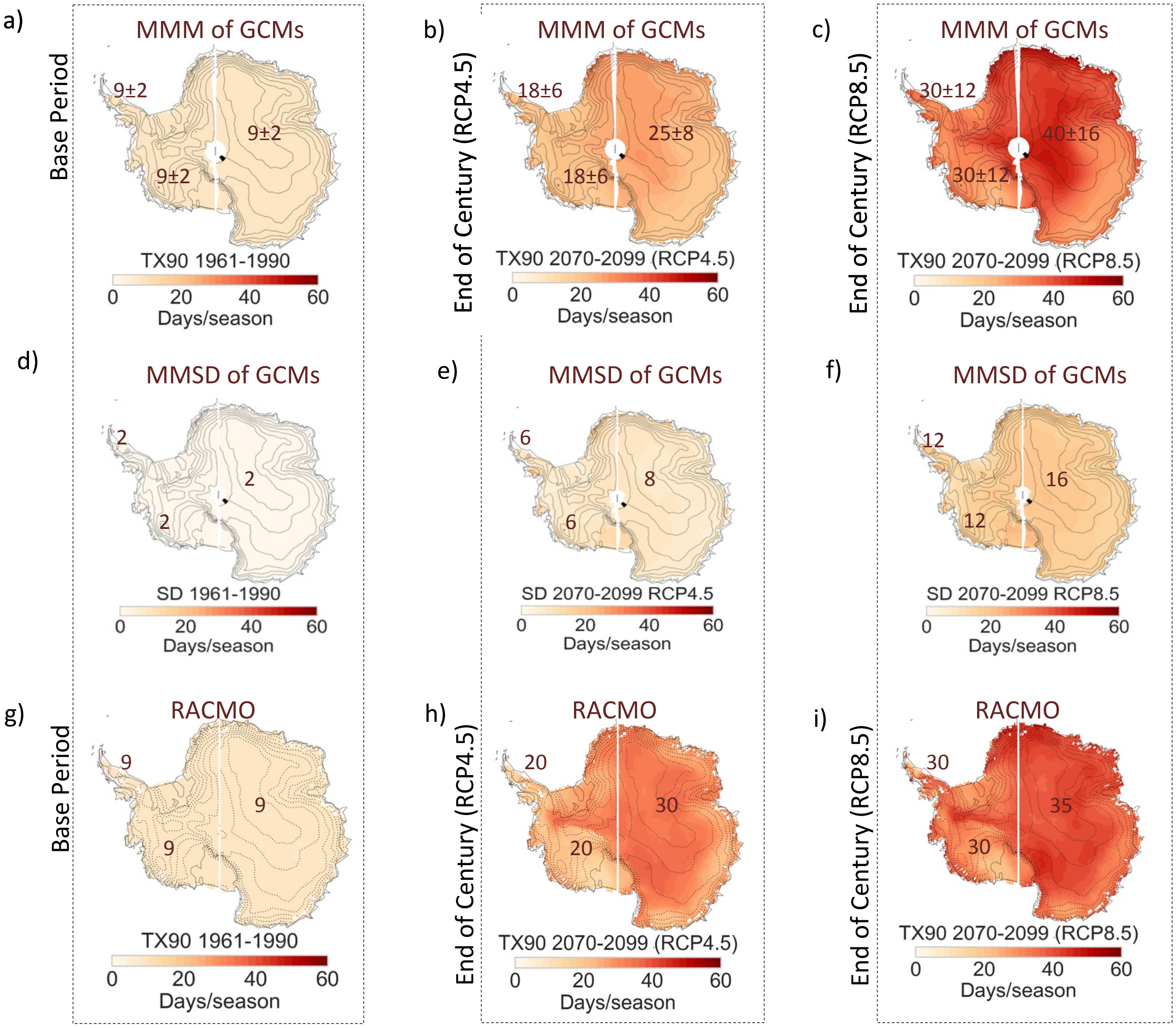

Fig. S7.
Number of "very warm" DJF days (TX90) over the base period 1961-1990 (1st column), over the period 2070-2099 under the RCP4.5 scenario (2nd column), and over the period 2070-2099 under the RCP8.5 scenario (3rd column).
a-c) Multi model mean (MMM) of TX90 estimates computed from the Global Climate Models (GCMs).
d-f) Multi model standard deviation (MMSD) of TX90 estimates computed from the GCMs.
g-i) TX90 estimates computed from the RACMO2 Regional Climate Model.
Plots were generated by using Python's Matplotlib Library[44].

Table S1: Global Climate Models (GCMs) considered in this study.

Institute	Full model acronym	Resolution	Main reference
Max Planck Institute for Meteorology	MPI-ESM-LR	1.9° x 1.9°	Raddatz et al.[45]
Max Planck Institute for Meteorology	MPI-ESM-MR	1.9° x 1.9°	Raddatz et al.[45]
Canadian Centre for Climate Modelling and Analysis	CanESM2	2.8° x 2.8°	Chylek, P., et al.[46]
NOAA Geophysical Fluid Dynamics Laboratory	GFDL ESM2M	2.0° x 2.5°	Dunne et al.[47]
NOAA Geophysical Fluid Dynamics Laboratory	GFDL-CM3	2.0° x 2.5°	Delworth et al.[48]
NOAA Geophysical Fluid Dynamics Laboratory	GFDL-ESM2G	2.0° x 2.0	Dunne et al.[49]
Institute for Numerical Mathematics (INM)	INMCM4	1.5° x 2°	Volodin et al.[50]
JAMSTEC (Japan Agency for Marine-Earth Science and Technology)	MIROC-ESM-CHEM	2.8° x 1.7°	Watanabe et al.[51]
Model for Interdisciplinary Research on Climate	MIROC5	1.4° x 1.4°	Watanabe et al.[52]
Model for Interdisciplinary Research on Climate	MIROC-ESM	2.8° x 2.8°	Watanabe et al.[51]
Centro Euro-Mediterraneo per I Cambiamenti Climatici	CMCC-CM	2°x2°	Scoccimarro et al.[53]
Centro Euro-Mediterraneo per I Cambiamenti Climatici	CMCC-CMS	1.9° x 1.9°	Scoccimarro et al.[53]
Centre National de Recherches Meteorologiques - Centre Europeen de Recherche et Formation Avancees en Calcul Scientifique.	CNRM-CM5	1.4° x 1.4°	Voldoire et al.[54]
LASG-CESS Institute of Atmospheric Physics, Chinese Academy of Sciences and CESS,Tsinghua University	FGOALS-G2	2.8° x 2.8°	Li et al.[55]
NASA-GISS	GISS-E2-R	2.0° x 2.5°	Schmidt et al.[56]

Table S2. Center of the grid points (of the RACMO2 model) that exhibited the highest surface melt on each ice shelf over the period 1981-2010.

Ice Shelf	Latitude	Longitude
George VI	-72.5	-69.0
Wilkins	-70.0	-72.5
Larsen C	-66.5	-61.0
Abbot	-72.5	-101.5
Shackelton	-65.5	+96.5
West	-66.5	+86.5
Amery	-69.0	+73.5
Baudouin	-69.5	+31.5
Fimbul	-70.0	-0.5
Ronne	-75.5	-60.5
Ross	-78.5	-159.5

References

45. Raddatz T. *et al*. Will the tropical land biosphere dominate the climate-carbon cycle feedback during the twenty first century? *Clim. Dynam.* **29**, 565-574 (2007).

46. Chylek P. *et al*. Observed and model simulated 20th century Arctic temperature variability: Canadian earth system model CanESM2. *Atmos. Chem. Phys.* **11**(8), 22893-22907 (2011)

47. Dunne J.P. *et al*. GFDL's ESM2 global coupled climate-carbon Earth System Models Part II: Carbon system formulation and baseline simulation characteristics. *J. Clim.* **26**, 7 (2013).

48. Delworth T.D. *et al*. GFDL's CM2 global coupled climate models-Part I: Formulation and simulation characteristics *J. Clim.* **19**, 643-674 (2006).

49. Dunne J. P. *et al*. GFDL's ESM2 global coupled climate–carbon earth system models. Part I: Physical formulation and baseline simulation characteristics. *J. Clim.* **25**(19), 6646-6665 (2012).

50. Volodin E. M., Dianskii N. A. & Gusev A. V. Simulating present-day climate with the INMCM4. 0 coupled model of the atmospheric and oceanic general circulations. IZV. *Atmos. Ocean. Phys.* **46**(4), 414-431 (2010).

51. Watanabe S. *et al*. MIROC-ESM: Model description and basic results of CMIP5-20c3m experiments. *Geosci. Model Dev.* **4**(4), 845 (2010).

52. Watanabe S. *et al*. Improved climate simulation by MIROC5: mean states, variability, and climate sensitivity. *J. Clim.* **23**(23), 6312-6335 (2010).

53. Scoccimarro E. *et al*. Effects of Tropical Cyclones on Ocean Heat Transport in a High Resolution Coupled General Circulation Model. *J. Clim.* **24**, 4368-4384 (2011).

54. Voldoire A. *et al*. The CNRM-CM5.1 global climate model: description and basic evaluation. *Clim. Dynam.* ***40***(9), 2091-2121 (2012).

55. Li L. *et al*. The flexible global ocean-atmosphere-land system model, Grid-point Version 2: FGOALS-g2. *Adv. Atmos. Sci.* **30**(3), 543-560 (2013).

56. Schmidt G.A. *et al*. Present day atmospheric simulations using GISS ModelE: Comparison to in-situ, satellite and reanalysis data *J. Clim.* **19**, 153-192 (2006).
